# Serum Soluble CD89-IgA Complexes Are Elevated in IgA Nephropathy without Immunosuppressant History

**DOI:** 10.1155/2020/8393075

**Published:** 2020-01-16

**Authors:** Haiting Wu, Xiaoyan Wang, Zhe Yang, Qing Zhao, Yubing Wen, Xuemei Li, Wei Zhang, Ruitong Gao

**Affiliations:** ^1^Division of Nephrology, Department of Internal Medicine, Peking Union Medical College Hospital, Chinese Academy of Medical Sciences & Peking Union Medical College, Beijing 100730, China; ^2^Department of Immunology, School of Basic Medicine, Institute of Basic Medical Sciences, Chinese Academy of Medical Sciences and Peking Union Medical College, Beijing 100005, China; ^3^Department of Clinical Laboratory, Key Laboratory of Cancer Prevention and Therapy, National Clinical Research Center of Cancer, Tianjin Medical University Cancer Institute and Hospital, Tianjin 300060, China

## Abstract

**Purpose:**

CD89 (Fc*α*RI), the receptor of IgA, can shed from cells to form complexes with IgA in serum and is supposed to participate in the pathogenesis of IgA nephropathy (IgAN). There are contradictory results on their utility in clinical practice. This study is aimed at investigating whether sCD89-IgA complexes can help in the diagnosis or evaluation of the disease.

**Methods:**

A sandwich ELISA was established using anti-CD89 as a capture antibody and HRP-conjugated anti-IgA as a detection antibody. This method was used to measure serum levels of sCD89-IgA complexes in IgAN patients without immunosuppressant history and healthy subjects. Correlations between serum levels of sCD89-IgA complexes and disease severity were analyzed.

**Results:**

Serum sCD89-IgA complexes increased with age (*P* < 0.001). IgAN patients had higher sCD89-IgA complex levels compared with age- and gender-matched normal healthy individuals (*P* < 0.001). IgAN patients had higher sCD89-IgA complex levels compared with age- and gender-matched normal healthy individuals (*P* < 0.001). IgAN patients had higher sCD89-IgA complex levels compared with age- and gender-matched normal healthy individuals (

**Conclusions:**

Serum sCD89-IgA complexes can guide diagnosis of IgAN in patients without immunosuppressant history, but provide limited help in clinicopathologic prediction.

## 1. Introduction

IgA nephropathy (IgAN) is the most common primary glomerular disease in the world and is a major cause for end-stage renal failure [1]. One of the characteristics of IgAN is deposition of polymeric IgA1 in the mesangial regions in the kidney [2]. The cause of the deposition is still under debate. Nevertheless, recurrence of IgA deposits in renal grafts of IgAN patients [3] is a compelling evidence suggesting that the kidney itself is an innocent bystander and circulating IgA or its related complexes play an important role in the pathogenesis of IgAN [4].

The IgA Fc receptor (CD89/Fc*α*RI) is expressed on myeloid cells including monocytes, macrophages, neutrophils, eosinophils, and dendritic cells [5]. The receptor can be enzymatically cleaved from the cell membrane and forms soluble receptor (sCD89) in circulation, which covalently linked to IgA [6]. Serum sCD89-IgA complexes may participate in the pathogenesis of IgAN according to animal experiments [7, 8]. However, earlier data shows that sCD89-IgA complexes are not specific for IgAN compared with healthy volunteer [5]. The application of serum sCD89-IgA complex concentration is undetermined in IgAN. Evidence is extremely limited in Asian population. In present research, we tried to explore the clinical implication of sCD89-IgA levels in Chinese IgAN patients and healthy subjects.

## 2. Subjects and Methods

### 2.1. Subjects

The 30 serum samples (14 males and 16 females) of biopsy-proved IgAN patients were obtained from the Division of Nephrology, Department of Internal Medicine, Peking Union Medical College Hospital (PUMCH). None of the patients had clinical or laboratory evidence of underlying systemic diseases such as systemic Henoch-Schönlein purpura, chronic liver diseases, chronic infectious diseases, connective tissue diseases, or chronic inflammatory bowel disease. None of the patients had immunosuppressive therapy history. The serum of 254 healthy controls (100 males and 154 females) were obtained from PUMCH. All the subjects included were Chinese Han race. All serum samples were stored in -80°C before use. This study was approved by the local Ethical Committee.

### 2.2. Clinical, Biochemical, and Histologic Data

Laboratory tests of the patients including serum creatinine, cystatin C, albumin, hypersensitive C-reactive protein (hsCRP), urinary routine test, and 24-hour proteinuria were measured at the same period as the sCD89-IgA complex tests. 24 patients underwent renal biopsy in PUMCH, while the other 6 in other hospitals. All the renal specimens from PUMCH were taken within 1 week after sCD89-IgA complex tests and graded by the Oxford Classification 2016 [9]. Serum creatinine, 24-hour proteinuria, and medications during each follow-up were recorded. In the last follow-up, rapid progression was confirmed by definition of average decline of estimated glomerular filtration rate (eGFR) more than 5 mL/min/1.73^2^/year.

### 2.3. Antibodies

Mouse anti-CD89 mAbs (MIP8a, MIP15b, MIP38c, and MIP71a) were developed as described before [10] and purified using protein A columns. Anti-IgA antibodies, KT40 and KT41, were from Absea Biotechnology Ltd. (Beijing, China).

### 2.4. Measuring sCD89-IgA Complexes

Ninety-six-well microtiter plates were coated overnight at 4°C with 100 *μ*L (10 *μ*g/mL) of mouse anti-CD89 mAbs (MIP8a, MIP15b, MIP38c, and MIP71a) in 50 mM carbonate-bicarbonate buffer, pH 9.6. The plates were washed with phosphate-buffered saline containing 0.05% Tween 20 (PBST) three times and blocked with 200 *μ*L/well of 2% bovine serum albumin for 2 h at room temperature. Then serum samples diluted in the blocking buffer were added and incubated for 2 h at room temperature. After washing, HRP-conjugated KT40 was added and incubated for 1 h at room temperature. The color was developed using ABTS (Amresco, USA) as a substrate and the absorbance was measured at 405 nm with a microplate reader (BioTek Synergy 4).

### 2.5. Immunoprecipitation Analysis of sCD89-IgA Complexes Using MIP15b Beads

To prepare sCD89-IgA complexes, 10 sCD89-free serum samples measured by ELISA were mixed and dispensed into 6 tubes (200 *μ*L/tube). Then, recombinant sCD89 was added into each tube with desired concentrations and the samples were incubated at 4°C overnight. Then, the serum samples were absorbed with 5 *μ*L MIP15b beads at 4°C for 4 h with rotation. After being separated with serum by centrifugation through microcentrifuge spin columns (Pierce, Rockford, IL, USA), the beads were washed with PBS for three times. Then, sCD89-IgA complexes were eluted with 50 *μ*L of 0.1 M glycine (pH 2.5) and were immediately neutralized by 2 M Tris-Cl (pH 8.0).

### 2.6. Western Blot Analysis

All of the eluates from MIP15b beads were run on 12% SDS-PAGE under reducing conditions. The proteins were then transferred onto nitrocellulose membranes. After blocking with 5% skimmed milk for 1 h at room temperature, the membranes were cut from 46 kDa position into two pieces. The one with molecular weight smaller than 46 kDa was incubated with MIP8a and MIP15b anti-human CD89 at 4°C overnight. After washing 5 times with PBST, the membranes were incubated with HRP-conjugated goat anti-mouse IgG (Sigma) for 1 h at room temperature. The membrane with molecular weight bigger than 46 kDa was incubated with HRP-conjugated KT13 anti-human IgA (Absea) for 1 h at room temperature. Then, the membranes were washed and the bands were visualized by enhanced chemiluminescence (Pierce).

### 2.7. Measuring Serum IgA Concentrations

Ninety-six-well microtiter plates were coated with 2 *μ*g/mL of KT41 in carbonate-bicarbonate buffer, pH 9.6. After washing and blocking, serum samples diluted in 1 : 6000 were added and incubated for 1 h at room temperature. Then, the plates were washed and HRP-conjugated KT40 was added and incubated for 1 h at room temperature. The color was developed using ABTS as a substrate and the absorbance was measured at 405 nm.

### 2.8. Statistical Analyses

Continuous variables were expressed as the mean ± standard deviation or median (interquartile range), and categorical variables were expressed as absolute numbers. The Mann-Whitney test or Kruskal-Wallis test was used to analyze differences between two groups. Spearman's rank correlations were used for calculation of correlations between data with nonnormal distribution. Receiver operating characteristic (ROC) curve was constructed to evaluate the predictive power for the diagnosis of IgAN. Statistical analysis was performed using GraphPad Prism 5.0 (GraphPad Software Inc., San Diego, CA) and SPSS statistical software version 23.0 (SPSS Inc., Chicago, IL). *P* < 0.05 was considered significant.

## 3. Results

### 3.1. sCD89-IgA Complexes in Serum Can Be Detected by Sandwich ELISA and Immunoprecipitation

**Figure 1:**
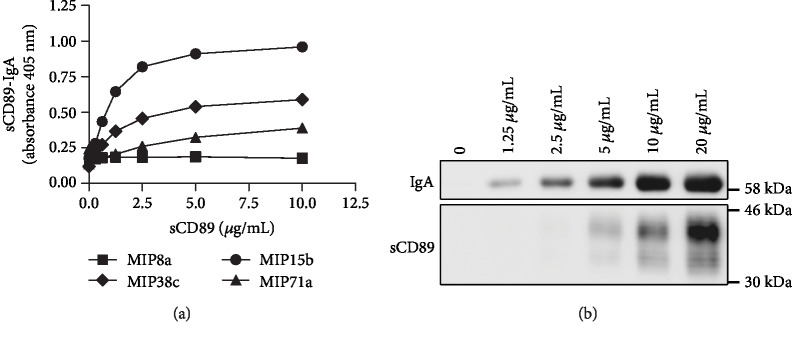
Measurement of serum sCD89-IgA complexes. (a) Detection by sandwich ELISA. Microtiter plates were coated with mouse anti-CD89 IgG (MIP8a, MIP15b, MIP38c, or MIP71a, respectively). Recombinant sCD89 was added into sCD89-free serum at indicated concentrations. The bound sCD89-IgA complexes were detected by HRP-conjugated anti-IgA antibody, KT40. (b) Detection of sCD89-IgA complexes by Western blotting. Recombinant sCD89 was added into the sCD89-free serum at indicated concentrations. After incubation at 4°C overnight, sCD89-IgA complexes were pulled down by MIP15b-beads. The sCD89-IgA complexes absorbed on the beads were run on SDS-PAGE, and IgA and CD89 were analyzed separately by Western blotting (see Subjects and Methods).

To quantify sCD89-IgA complexes in serum, we developed a sandwich ELISA with monoclonal anti-CD89 antibodies, MIP8a, MIP15b, MIP38c, and MIP71a. MIP8a-coated plates failed to detect sCD89-IgA complexes, which was because MIP8a was a neutralizing antibody and its epitope was shaded when CD89 bound to IgA. The rest of the monoclonal antibodies could detect sCD89-IgA complexes at various sensitivities (Figure 1(a)). MIP15b was the best among all the antibodies. It could detect sCD89-IgA complexes in a dose-dependent manner with a detection limit of 116 ng/mL. sCD89-IgA complexes in serum could also be pulled down by beads conjugated with MIP15b. Western blotting showed that MIP15b could capture sCD89 in the serum and coprecipitate IgA that bound to sCD89 (Figure 1(b)).

### 3.2. Serum sCD89-IgA Levels Are Elevated in IgAN Patients

**Figure 2:**
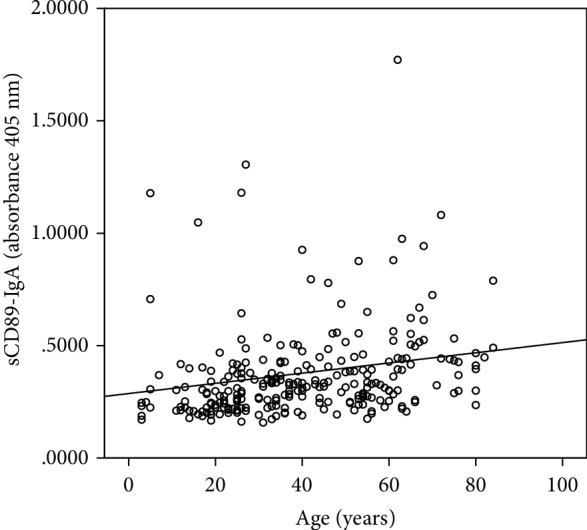
Serum sCD89-IgA complexes levels increased with age (*P* < 0.001).

**Figure 3:**
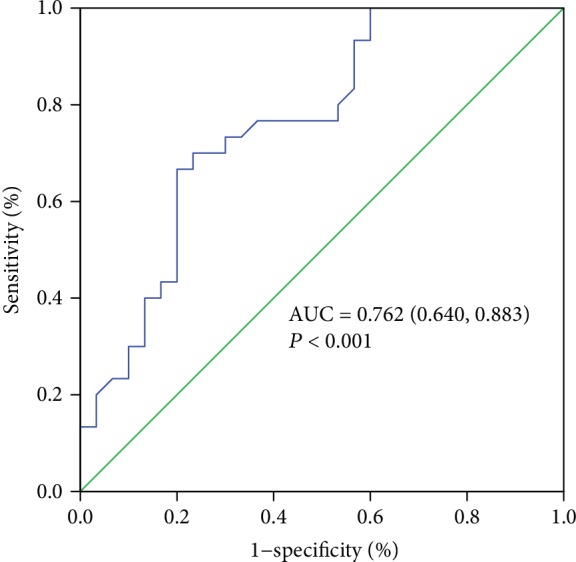
Receiver operating characteristic (ROC) curve of the serum sCD89-IgAN showing sensitivity and specificity for the diagnosis of IgAN comparing the IgAN group (*n* = 30) versus the healthy group (*n* = 30).

sCD89-IgA can be detected in healthy individuals, and the level increased with age (Figure 2, *r* = 0.373, *P* < 0.001). sCD89-IgA complexes in IgAN patients were 1 : 1 matched by age and gender with healthy controls. A significant increase of sCD89-IgA complexes was found in IgAN patients compared to normal controls (*P* < 0.001, Table 1). Serum IgA levels of IgAN patients were also significantly different from the control group (*P* = 0.002). To assess the predictive value of serum sCD89-IgAN for predicting IgAN, ROC curve analysis was performed. Serum sCD89-IgAN predicted IgAN (AUC = 0.762 (0.640-0.883), *P* < 0.001, Figure 3), with a sensibility of 66.7% and specificity of 80.0% at a cutoff value of 0.353 (OD405nm). Other clinical and pathological characteristics of the IgAN patients were also listed in Table 1.

### 3.3. Serum sCD89-IgA Were Not Correlated with Clinicopathologic Characteristics in IgAN Patients

Baseline serum IgA level increased with sCD89-IgA significantly (*P* < 0.001), but no other baseline clinicopathlogic characteristics showed correlation with sCD89-IgA complexes in IgAN patients. After CD89-IgA complexes tests, 15 (50%) patients received glucocorticoid, 10 (33.3%) were treated with immunosuppressants including cyclophosphamide, cyclosporin, mycophenolate mofetil, azathioprine, and tripterygium glycosides. The average eGFR decline rate was 2.69 ± 3.75 mL/min/1.73m^2^/year. One patient entered end-stage renal failure. sCD89-IgA cannot predict rapid progression of IgAN (Table 2).

## 4. Discussion

In this study, we found that sCD89-IgA complex levels were related with age, which is in concordance with Jhee et al. [11]. It is well known that aging is associated with a dysregulation of the immune system including cellular and molecular alterations [12], and aging may lead to decreased clearance of complexes. So we compared sCD89-IgA complexes in IgAN with age- and gender-matched controls. We found that IgAN patients had increased sCD89-IgA complex levels compared with normal controls, which was in agreement with Launay et al.'s observations [7]. However, Boog et al. reported sCD89-IgA complexes were not specific in levels or size distribution between IgAN patients and healthy volunteers [5]. Vuong et al. also reported that nonprogressive IgAN had similar levels of sCD89-IgA complexes with healthy subjects, and progressive IgAN group had even lower levels compared with the nonprogressive group [13]. This discrepancy led us to presume possibilities. Firstly, all the patients included in our study received no steroid or immunosuppressants before testing sCD89-IgA complexes. Berthelot et al. reported a group of recurrent IgAN after transplantation [14]. Serum sCD89-IgA complex levels increased after recurrence, but decreased six months after steroid pulse, which implied sCD89-IgA complexes were involved in the disease activity and can be suppressed by treatment. Boog et al. and Vuong et al. did not provide information about therapy strategy before the sCD89-IgA complex tests. And we supposed, in Vuong et al.'s research, the progressive group might have received more aggressive treatment than the nonprogressive group, which could partly explain the lower concentration of sCD89-IgA complexes. Since patients in our study had no history of immunosuppressive treatments, we think the result can represent baseline characteristic. Secondly, sCD89 detected in a different study might be different. At least two isoforms of sCD89 exist in vivo. Launay et al. described heavily glycosylated CD89 molecules (50–70 kD) in polyethylene glycol precipitates with sandwich ELISA [7]. Boog et al. found 30 kD solute CD89 molecules with Western blot and dot blot [5]. Jhee et al. tested both isoforms with different antibodies by ELISA [11]. And as reported previously, sCD89 was identified as 28-36 kD molecules, with a 25 kD backbone in our laboratory [15]. The relationship and differences between the two isoforms are still unclear. Then, the discrepancy of different levels of sCD89-IgA complexes among researches may be partly explained by different isoforms tested.

In the present study, although sCD89-IgA complexes were found elevated in IgAN patients, their levels had no correlation with clinical manifestations and oxford classification. Baseline sCD89-IgA complexes also cannot distinguish rapid progressive patients. Jhee et al. found a week negative association between sCD89-IgA complexes and renal function, which might imply the potential role in the disease progression. But the authors pointed that this tendency could be a result of decreased clearance of the complexes. And further multivariate analysis showed no significant relation between these two factors. They also found that sCD89-IgA did not predict decline in eGFR > 30% [11]. To date, there is little evidence supporting serum sCD89-IgA complexes in evaluation or progress prediction of IgAN. Only one study examined urine CD89 in IgAN and Henoch-Schönlein purpura nephritis [16]. Urinary CD89 after adjusting by proteinuria and urinary creatinine was significantly lower in patients with active IgAN/HSPN compared to those in complete remission. The author explained that this could be because of the deposition of CD89 in the kidney in active patients. However, more research is needed to confirm the results.

The underlying roles of sCD89-IgA complexes in IgAN are still undetermined. In the present study, the complex level positively correlated with serum IgA, which was in accordance with a large sample size Asian research [11]. The elevation of IgA serum level is a common feature in IgAN [17], which might be a reaction to systemic or chronic mucosal stimulation, such as a persistent microbial infection or incapable of adequate elimination [18]. And aberrant mucosal immunity is thought to be important in IgAN pathogenesis. Then, elevation of sCD89-IgA complexes in IgAN might be only a reflection of elevated serum IgA. However, animal experiments showed some evidence of pathogenicity of sCD89-IgA complexes. The complexes can be detected in the sera of human CD89 transgenic mice which spontaneously develop IgAN, and sCD89-injected mice can develop mesangial IgA1 deposition [7]. Mesangial sCD89-IgA1 deposition has also been revealed in IgAN patients [8].

This study has several limitations. Firstly, we did not analyze whether molecular structures of sCD89-IgA complexes are different between IgAN and heathy individuals. Whether aberrant glycosylated IgA molecules are more likely to be covalently linked with CD89 structure will be explored in further experiments. Secondly, we only detected sCD89-IgA complexes at the baseline, concentration trend or time-average sCD89-IgA complexes may provide more information, especially for long-term monitor. Thirdly, we did not perform CD89-IgA complex staining in the kidney. So whether the higher serum complexes levels lead to renal deposition is unproven in pathology. Finally, the study is based in a single center, and the sample size is relatively small which limit further analysis.

In summary, our results indicate that serum sCD89-IgA complexes can guide the diagnosis of IgAN in patients without immunosuppressant history, but have little help in predicting clinicopathologic characteristics or progression rate. More studies are needed in researching the relationship between different sCD89 isoforms and their pathological impact.

## Figures and Tables

**Table 1:** Clinical and laboratory findings of IgAN patients and healthy controls.

	IgAN (*n* = 30)	Healthy controls (*n* = 30)	*P* value
Age (years)	34 ± 10	32 ± 10	0.388
Gender (M/F)	14/16	13/17	0.795
IgA (mg/mL)	3.14 (2.40, 4.08)	2.12 (1.46, 2.82)	0.002
sCD89-IgA (OD405nm)	0.401 (0.290, 0.556)	0.266 (0.217, 0.351)	<0.001
Creatinine (*μ*mol/L)	72 (56, 85)		
eGFR (mL/min/1.73m^2^)	104.6 (82.6, 118.9)		
Cys-C (mg/dL)	0.91 (0.76, 1.11)		
Albumin (g/L)	42.3 ± 6.6		
hsCRP(mg/L)	0.64 (0.40, 1.31)		
Proteinuria (g/24 h)	1.00 (0.51, 3.02)		
U-RBC (-/1+/2+/3+)	2/5/4/18		
Pathology (Oxford classification)^a^			
M0/M1	0/24		
E0/E1	8/16		
S0/S1	8/16		
T0/T1/T2	20/2/2		
C0/C1/C2	2/19/3		
RASI (yes/no)	20/10		

^a^Renal histological grading was obtained in 24 patients who underwent biopsy in Peking Union Medical College Hospital. RASI: renin angiotensin system inhibitor; F: female; M: male; Cys-C: Cystatin C; U-RBC: urinary red blood cell.

**Table 2:** Association of serum sCD89-IgA complexes with clinicopathologic characteristics.

	*r* or *Z* or *χ*^2^	*P*
Serum creatinine	0.041	0.829
eGFR	-0.278	0.137
Cys-C	0.020	0.935
Albumin	0.270	0.149
24-hour proteinuria	0.068	0.722
hsCRP	0.105	0.650
Serum IgA	0.555	<0.001
U-RBC	1.451	0.694
Pathology (Oxford classification)		
E	-0.796	0.452
S	-0.919	0.383
T	3.084	0.214
C	4.696	0.096
ACEI (before sCD89-IgA complex test)	-0.044	0.983
Glucocorticoid (after sCD89-IgA complex test)	-0.956	0.335
eGFR decline rate	0.254	0.231
Rapid progression	-0.349	0.757

eGFR: estimated glomerular filtration rate; Cys-C: cystatin C; hsCRP: hypersensitive C-reactive protein; IgAN: IgA nephropathy; U-RBC: urine red blood cell; ACEI: angiotensin converting enzyme inhibitor.

## Data Availability

The data used to support the findings of this study are available from the corresponding authors upon request.
